# Human papilloma virus (HPV) infection is associated with HIV-1 infection and AIDS in HIV-infected adult patients from Zaria, Northern Nigeria

**DOI:** 10.11604/pamj.2013.15.38.2349

**Published:** 2013-05-31

**Authors:** Dimie Ogoina, Bolanle Olufunke Musa, Geoffrey Chukwubuike Onyemelukwe

**Affiliations:** 1 Department of Medicine, Niger Delta University, Wilberforce Island, Amassoma, Bayelsa State, Nigeria; 2 Department of Medicine, Immunology Unit, Ahmadu Bello University Teaching Hospital, Zaria, Kaduna State, Nigeria

**Keywords:** HPV, HIV-1, CD4 cell count, AIDS

## To the editors of the Pan African Medical Journal

Human papilloma virus (HPV) is the most common sexually transmitted virus and it is estimated that about 75% of sexually active women and men will acquire a genital HPV infection at some time.[1] There is an advance clinical association between HPV and HIV-1[2, 3] and it has been suggested that HPV may also facilitate progression of HIV-1 disease by recruitment of HIV target cells, such as CD4+ T-cells and macrophages, into the site of active HPV infection, and by inducing the production of inflammatory cytokines, including IL-6, TNF- 45; and IL-1, which in turn induce the replication and reverse transcription of HIV-1 [4].

In view of paucity of studies from Nigeria and in order to provide preliminary information on the clinical associations between HPV and HIV-1 infections, we conducted a cross sectional study between May and July 2010 among 63 HIV-1 infected adults seen at Ahmadu Bello University Teaching Hospital Zaria (ABUTH), Kaduna State, Nigeria, and 26 HIV-negative apparently healthy adult controls living in Zaria, Northern Nigeria. After obtaining demographic and clinical data, including sexual history, we assayed serum IgG antibodies to HPV by ELISA (Weifang Kanghua Biotech Co. Ltd, China) and CD4 cell counts by flow cytometry. Ethical approval for the study was obtained for the institutional review board of ABUTH and all study participants gave consent for the study. Data was analysed using SPSS 17. For all analyses, P<0.05 was taken as statistically significant.

Of the 63 HIV-infected patients, 40 (63.5%) were females, 55 (87.4%) were ever married and 15 (23.8%) had 3 or more lifetime sexual partners. Of the 26 HIV negative controls, 13 (50%) were females, 17 (65.4%) were never married, and 19 (73.1%) had 1 to 2 lifetime sexual partners while 7 (28%) had no previous sexual intercourse. The mean ages and standard deviation (Ranges) of the HIV positive and negative study participants were 36 ± 8.6years (20-57years) and 34 ± 10.7 years (21-56 years) respectively, (p > 0.05, student's t test).

With regard to IgG HPV antibody serostatus, 1(3.8%) of the 26 HIV-negative participants and 26 (41.3%) of the 63 HIV-positive patients were HPV IgG seropositive. The HIV-positive patients were about 18 times more likely to be HPV seropositive than the HIV-negative adults (OR 17.6, 95% CI 2.2-138, p=0.0006). The only HIV-negative HPV seropositive participant was a 28year old male single civil servant who had two lifetime sexual partners. Among HIV-infected patients, univariate and multivariate (using logistic regression) analyses (Table 1), revealed that CD4 cell count was the only independent variable associated with HPV seropositivity. Patients with CD4<200cells/ul (indicative of AIDS) had 5 times more likelihood of been HPV IgG seropositive than those with CD4 cell count ≥200cells/ul (0R 5.1, 95% CI 1.3-20.8, p=0.022). Only three (11.5%) of the 26 HPV seropositive patients had clinical evidence of anogenital and facial warts. Papanicolaou (Pap) smears were not done.

**Table 1:** Associations Between HPV IgG Seropositivity And Clinical Variables Of HIV-Infected Adults from Zaria, Northern Nigeria

Variable	Univariate analyses				Multivariate analyses		
	N (%)	OR	95% CI	P value	Adjusted OR	95% CI	P value
**Age group (yrs)**							>0.05
18-40*	17 (37.8)	1	1	>0.05	1	0.3-4.1	
>40	9 (50)	1.7	0.6-5.0		1.1		
**Gender**							>0.05
Male	10(43.5)	1.2	0.4-3.3	>0.05	0.86	0.2-3.2	
Female*	16 (40)	1			1	1	
**Marital status**							>0.05
Never married*	2 (25)	1	1	>0.05	1	1	
Ever married	24 (43.6)	2.3	0.4-12.5		3.4	0.5-23.4	
**Lifetime number of sexual partners**							>0.05
1-3*	19 (39.6)	1	1	>0.05	1	1	
>3	7(46.7)	1.3	0.4-4.3		1.8	0.4-8.1	
**HIV clinical stage**							>0.05
Early HIV (stage 1/2)*	7 (35)	1	1	>0.05	1	1	
Late HIV (stage 3/4)	19 (44.2)	1.5	0.5-4.4		1.1	0.3-3.6	
**CD4 category (cells/ul)**							0.022
CD4 ≥ 200*	16 (33.3)	1	1	0.02	1	1	
CD4<200	10 (66.7)	4.0	1.2-13.7		5.1	1.3-20.8	

* NB:=reference variable, N=number, OR=odds ratio, CI=confidence interval, p >0.05=not significant

This study from Zaria, Northern Nigeria has shown that cumulative HPV infection detected by assay of serum IgG antibodies to HPV occurs more frequently in HIV-infected patients than in HIV-negative healthy adults. This finding is in agreement with studies from other African countries [5–7], and may be attributed to poor clearance of HPV infection in HIV-infected patients relative to HIV-negative adults [3, 8] and the fact that both HIV and HPV infections share similar route and risk factors for infection [2]. It is noteworthy that IgG HPV seropositivity was independently associated with features of advanced immunosuppresion or AIDS (CD4<200cells/ul). This finding may be due to the positive correlation between immune status and HPV clearance, as patients with significant immunosuppresion are less likely to clear HPV infection and consequently develop persistent HPV infection with continually detectable HPV antibodies [3, 8]. Alternatively, it is probably that HPV infection facilitated the progression of HIV to AIDS in our patient through mechanisms previously described [4]. In agreement with our findings, various prospective and cross sectional studies from other parts of the world have also shown that among HIV-1 infected patients, active, chronic and persistent HPV infection is more common in those with features of significant immunosuppresion AIDS defined as CD4<200cells/ul [3, 6, 7, 9].

In Nigeria, there are more than 3.1 million HIV-infected people [10] and about 23.7% of women and 73% of men of the general population harbour HPV genital infection [11]. In view of the high rates of both HPV and HIV infection in Nigeria, it is necessary for future prospective studies to be undertaken in Nigeria using larger sample sizes and more specific assays, such as assay of high risk HPV serotypes and HPV DNA, to shed further light on the associations between HPV and HIV/AIDS.

In conclusion, cumulative HPV infection is high in HIV-infected patients from Zaria, Northern Nigeria, especially among AIDS patients. These findings support the need for routine and early screening of all HIV infected patients for HPV infection in Nigeria, as well as routine clinical evaluation of all HIV-infected patients for HPV-related manifestations.
